# How to Optimize Tuberculosis Health Education in College Under the New Situation? Based on a Cross-Sectional Study Among Freshmen of a Medical College in Guangxi, China

**DOI:** 10.3389/fpubh.2022.845822

**Published:** 2022-03-24

**Authors:** Tengyan Wu, Huimin He, Suosu Wei, Jian Pan, Jingjuan Yang, Shi Huang, Shijie Gan, Chengpeng Ye, Haiying Huo, Zhong Tang, Qiming Feng

**Affiliations:** ^1^Department of Health Service Management, School of Information and Management, Guangxi Medical University, Nanning, China; ^2^Editorial Board of Chinese Journal of New Clinical Medicine, The People's Hospital of Guangxi Zhuang Autonomous Region, Nanning, China

**Keywords:** tuberculosis, health education, KAP, freshmen, investigation

## Abstract

**Background:**

China is a country with a high burden of tuberculosis (TB), and students are the high-risk group for TB. The enrollment scale of colleges has increased dramatically due to the advancement of the enrollment expansion system of Chinese colleges. Consequently, this has brought severe challenges to TB prevention and control in colleges. In 2017, a new TB control guide for schools was issued in China, which included the 8 core knowledge of TB. The target of the overall awareness rate on TB among population was “≥85%,” which was proposed by the “13th Five-Year” National TB Control Plan in China. The cognition of the 8 core knowledge of TB in the new guide among college students is crucial to achieve this target, but few studies on this have been reported. Based on the abovementioned new situation and the new guide, this study aimed to investigate and analyze the cognition, attitudes, and health education needs on TB among freshmen of a medical college in Guangxi province, and discuss how to optimize TB health education in colleges in China.

**Methods:**

A cross-sectional study was conducted among freshmen of a medical college in Guangxi, China. A self-designed questionnaire was used to conduct an on-site questionnaire survey. The data was entered in Epidata 4.4.2.1 and was analyzed using SPSS version 25.0. Including descriptive statistics and *t-*test, and the criterion for statistically significant difference was *p* < 0.05.

**Results:**

A total of 583 freshmen responded to the survey questionnaires. Regarding cognition about the 5 related knowledge of TB, 551 (94.5%) freshmen knew about the predilection site of TB, while 333 (57.1%), 328 (56.4%), 257 (44.1%), and 201 (34.5%) freshmen knew about the pathogen, the policies about free treatment, the designated hospitals, and the World TB Day, respectively. Regarding cognition on the 8 core knowledge of TB, the overall awareness rate among the freshmen is 73.3%(3,420/4,664); the awareness rate of the knowledge that “guarantee adequate sleep, reasonable diet, and strengthen physical exercise can reduce the incidence of TB” among them was the highest at 88.7% (517/583); and the awareness rate of the knowledge that “coughing or sputum expectoration occurred for more than 2 weeks should be suspected of infecting TB and seeking medical treatment in time” among them was the lowest at 47.5% (277/583). Whether students received health education on TB (T = 4.267, *p* = 0.000) and whether students heard of TB (T = 3.739, *p* = 0.000) are the main factors of cognition. Five hundred sixty-two (96.4%) and 565 (96.9%) freshmen were willing to learn and tell others about the knowledge of TB, respectively. Three hundred seventy (63.5%.) freshmen, the highest amount, were willing to accept TB health education in the forms of “website, Weibo, and WeChat.”

**Conclusion:**

The cognition on the 5 related knowledge of TB among freshmen is unbalanced, and the overall awareness rate of the 8 core knowledge of TB among freshmen still needs to be improved. Freshmen who have not heard of TB and have not received TB health education before enrollment are the key intervention groups. It is recommended that institutions make full use of modern multimedia technology, continuously optimize the health education forms, implement precise policies, and strengthen the theoretical and practical health education on TB from the initial entry of freshmen into colleges.

## Introduction

Tuberculosis (TB) is a chronic respiratory infectious disease that seriously endangers human health caused by Mycobacterium tuberculosis (MTB). The Global Tuberculosis Control Report issued by the World Health Organization (WHO) showed that an estimated 10.0 million people fell ill with TB in 2019, a number that has been very slowly declining in recent years. Particularly, men and women aged ≥15 years accounted for 88% of the people who developed TB (1).With this being the case, college students (18 ≤ aged ≤ 25 years) are obviously one of the most vulnerable groups infected with TB (2–4). Therefore, strengthening the TB prevention and control in colleges is of great significance to reduce the incidence of TB and achieve the global goal of WHO's End TB Strategy.

China is one of the 30 countries with the highest TB burden in the world, with about 833 thousand TB new cases accounting for 8.4% of the global incidence and ranking third worldwide in 2019 (1). The fifth national TB survey conducted in 2010 showed that the prevalence of active TB was 459/100,000 when compared with the prevalence of 466/100,000 in 2000. However, it is estimated that 44.5% of China's population were infected with TB, representing an enormous challenge to public health (5–7). In recent years, the number of college students is increasing due to the dramatic increase in expansion of the enrollment scale of Chinese colleges. Hence, the situation of TB prevention and control in colleges becomes more serious (8, 9). On college campuses, it is easy for a breakout of TB to occur if TB patients appear because of the high degree of person-to-person interactions and relatively crowded dormitory settings (10–13). Hence, TB prevention and control in schools has been incorporated into the national TB Control Plan. The new TB control guide for schools was issued in the year 2017 in order to respond to the challenge, strengthen health education, and improve the cognition on TB among students. It is essential to timely understand the cognition, attitude, and practice of TB among college students. There have been many relevant studies on the cognition on TB among college students, but the relevant reports in freshmen group based on the new guide and the new situation are few in China.

Guangxi Zhuang Autonomous Region is an underdeveloped southwestern region of China where the TB epidemic is relatively serious. Studies have shown that TB cases among students are mainly concentrated in high school and college students (14, 15). Hence, improving the cognition on TB among college students is an important measure to control the TB epidemic in colleges of Guangxi (12). However, there are few reports on the cognition on TB among college students, particularly among college freshmen in Guangxi. Based on the new TB control guide, this study carried out investigation among freshmen in a Medical Colleges of Guangxi in order to understand the cognition, attitude, and health education demands on TB among freshmen, discuss the countermeasures of improving the cognition on TB of students, and the possible ways of optimizing TB health education in colleges.

## Materials and Methods

### Study Area

This study was conducted in a medical college from November 1, 2019 to December 30, 2019. The school is located in the city of Nanning, the capital of Guangxi Zhuang Autonomous Region where the burden of TB is heavier. The school has formed a complete education and training system for bachelor's, master's, doctoral, and post-doctoral. It currently has 7 college disciplines including medicine, management, education, and so on. There are a total of 3,100 undergraduate freshmen enrolled in September 2019, of which the ratio of the number of clinical medicine and non-clinical medicine was about 2.5:1.

### Study Design

A cross-sectional study was conducted among the freshmen of a medical college in Guangxi. A stratified cluster random sampling method was adopted to select all the students that were from eight clinical medicine classes and four non-clinical medicine classes to conduct the survey. All participants signed an informed consent form.

### Data Collection

The data was collected using a self-designed questionnaire which was according to the relevant literature being issued by China for conducting an on-site questionnaire survey. The questionnaire was initially pre-tested among 20 freshmen with accordance to feedback. This group of freshmen were later excluded from the study. The questionnaire was distributed to the freshmen by trained investigators, and the responses were self-administered by the respondents.

The questionnaire was divided into four parts. The first part includes questions on participants' socio-demographic characteristics including gender, ethnicity, birthplace, whether you have heard of TB, whether you have received TB health education, etc. The second part focuses on the cognition and the ways of learning about the knowledge of TB, including the five related knowledge of TB (there are the World TB Day,the pathogens, the common incidence location, the related free policies, and the designated hospitals of TB) and the eight core knowledge of TB (the transmission route, suspected symptoms, prevention and treatment measures, etc.) that were formulated according to the “School TB Prevention and Control Work Regulations (the edition of 2017)” (16). The third part focused on the related attitudes toward TB, including the attitude toward learning and disseminating the knowledge of TB and the attitudes toward patients with TB. Lastly, the fourth part focused on the demand for the ways of TB health education that are being implemented in colleges. The questionnaire consisted of both multiple-choice questions with single and multiple answers.

### Indicator Definition

This study evaluated the cognition level of TB among respondents by calculating the following two indicators separately: ① Awareness rate of the single knowledge (%) = the number of people surveyed who answered a certain item correctly/the number of valid surveys^*^100%; and ② Total awareness rate of the eight core knowledge (%) = ∑ The number of items that answered correctly of each survey respondent/(number of valid surveys^*^8)^*^100%.

### Data Analysis

The questionnaire data was entered using the software EpiData3.1 (EpiData Association, Denmark) by double-entry and verify. The statistical analysis was performed with the software Spss25.0 IBM Corporation, Armonk, State of New York). The statistical description was mainly based on the composition ratio and rate, and the difference analysis adopts the *t-*test. A *p* < 0.05 was considered significant.

### Ethical Approval and Confidentiality

The ethical approval was provided by the Research Ethics Board of Health (REBH), Guangxi Medical University, China. In addition, administrative clearance was obtained from the college administration. All the participants signed an informed consent form before participating in this study. The survey data is protected using a password encrypted folder in the principal investigator's and co-investigators' computers.

## Results

### Socio-Demographic Characteristics

A total of 587 questionnaires were distributed and 583 valid questionnaires were returned. The valid questionnaire rate was 99.3% (583/587). Table 1 shows that in 583 freshmen, the average age was 18.4 years (SD.722; Range 18–21 years), 343 of whom are female (accounting for 58.8%), 413 of whom are of Han nationality (accounting for 70.8%), 402 of whom are in clinical classes (accounting for 69.0%), 343 of whom come from rural areas (accounting for 58.8%), 522 of whom have heard of TB before attending college (accounting for 89.6), and 309 of whom have received TB health education before attending college (accounting for 53.0%).

**Table 1:** Socio-demographic characteristics of study freshmen (*n* = 583).

**Variables**	**Frequency**	**Percentage**	**Variables**	**Frequency**	**Percentage**
Gender			Birthplace		
Male	240	41.2	Rural area	343	58.8
Female	343	58.8	Urban area	240	41.2
Nationality			Whether you heard of TB?		
Han nationality	413	70.8	Yes, I understand the relevant content	387	66.4
Zhuang nationality	148	25.4	Yes, I or those around have suffered TB	135	23.2
Others	22	3.8	No, I never heard of TB	61	10.4
Profession			Whether you received TB health education?		
Clinical medicine	402	69.0	Yes	309	53.0
Others	181	31.0	No	274	47.0

### The Cognition About the Knowledge of TB Among Freshmen

#### The Cognition About the 5 Related Knowledge of TB

Table 2 shows that in the cognition on the five related knowledge of TB among freshmen, 551(94.5%) freshmen knew about the predilection site of TB, the highest reported among the values, while 333 (57.1%), 328 (56.4%), 257 (44.1%), and 201 (34.5%) freshmen knew about the pathogen, the policies about free treatment, the designated hospitals, and the World TB Day, respectively. All four of which reported a relatively low sequentially.

**Table 2:** The cognition of freshmen about the five related knowledge of tuberculosis (*n* = 583).

**Knowledge**	**Numbers of whom answered correctly (n)**	**Awareness rate (%)**
1. World TB Day.	201	34.5
2. The pathogene of TB.	333	57.1
3. The predilection site of TB.	551	94.5
4. The policies about free treatment for TB.	329	56.4
5. The designated hospitals for TB.	257	44.1

#### The Cognition About the Eight Core Knowledge of TB

Table 3 shows that in the cognition on the eight core knowledge of TB, the overall awareness rate among the freshmen is 73.3% (3,420/4,664). Meanwhile, the awareness rate of the knowledge that “guarantee adequate sleep, reasonable diet and strengthen physical exercise can reduce the incidence of TB” among them was the highest at 88.7% (517/583), and the awareness rate of the knowledge that “coughing or sputum expectoration occurred for >2 weeks should be suspected of infecting TB and seeking medical treatment in time” among them was the lowest at 47.5% (277/583).

**Table 3:** The cognition about the eight core knowledge of TB among freshmen (*n* = 583).

**Knowledge**	**Numbers of whom answered correctly (n)**	**Awareness rate (%)**
1. TB is a chronic infectious disease that seriously endangers the health of people for a long time.	328	56.3
2. TB is mainly transmitted through the respiratory tract, and everyone is likely to be infected.	479	82.2
3. Coughing or sputum extraction that occurred for more than 2 weeks should be suspected of infecting TB and seek medical treatment in time.	277	47.5
4. Do not spit anywhere, cover your mouth and nose when coughing or sneezing, and wearing a mask can reduce the spread of TB.	458	78.6
5. Standardize the whole course of treatment, the vast majority of TB patients can be cured.	397	68.1
6. It should be reported initiative that students with symptoms of TB or being diagnosed with TB at school.	477	81.8
7. Opening windows for ventilation can prevent TB effectively.	487	83.5
8. Ensuring adequate sleep, a reasonable diet and strengthening physical exercise can reduce the incidence of TB.	517	88.7
Total	3,420	73.3

### The Factors Associated With the Total Awareness Rate of the Eight Core Knowledge of TB

The *t-*test is used to analyze the factors associated with the total awareness rate of the eight core knowledge of TB among freshmen. Table 4 shows that whether students received health education on TB (*t* = 4.267, *p* = 0.000) and whether students have heard of TB (*t* = 3.739, *p* = 0.000) are influencing factors. Freshmen who have heard of TB and received TB health education before enrollment have a higher awareness rate of the 8 core knowledge of TB.

**Table 4:** The factors associated with the total awareness rate of the eight core knowledge of TB.

**Factors**	**Numbers of entries that are answered correctly (n)**	**The total awareness rate (%)**	***T*-value**	***P*-value**
Gender			−1.662	0.097
Male (*n* = 240)	1,375	71.6		
Female (*n* = 343)	2,045	74.5		
Professional			1.35^*^	0.178
Clinical Medicine (*n =* 402)	2,385	74.2		
Others (*n =* 181)	1,035	71.5		
Birthplace			−0.661	0.509
Rural area (*n =* 343)	1,999	72.8		
Urban area (*n =* 240)	1,421	74.0		
Ethnicity			0.178	0.859
Han nationality (*n =* 413)	2,426	73.4		
Others nationality (*n =* 170)	994	73.1		
Whether you heard of TB?			4.267	0.000
Yes (*n =* 522)	3,114	75.4		
No (*n =* 61)	306	62.7		
Whether you received TB health education?			3.739	0.000
Yes (*n =* 309)	1,887	76.3		
No (*n =* 274)	1,533	69.9		

* *Means the variance is not uniform, and the value of T' is taken*.

### The Ways of Obtaining the Knowledge of TB

**Figure 1:**
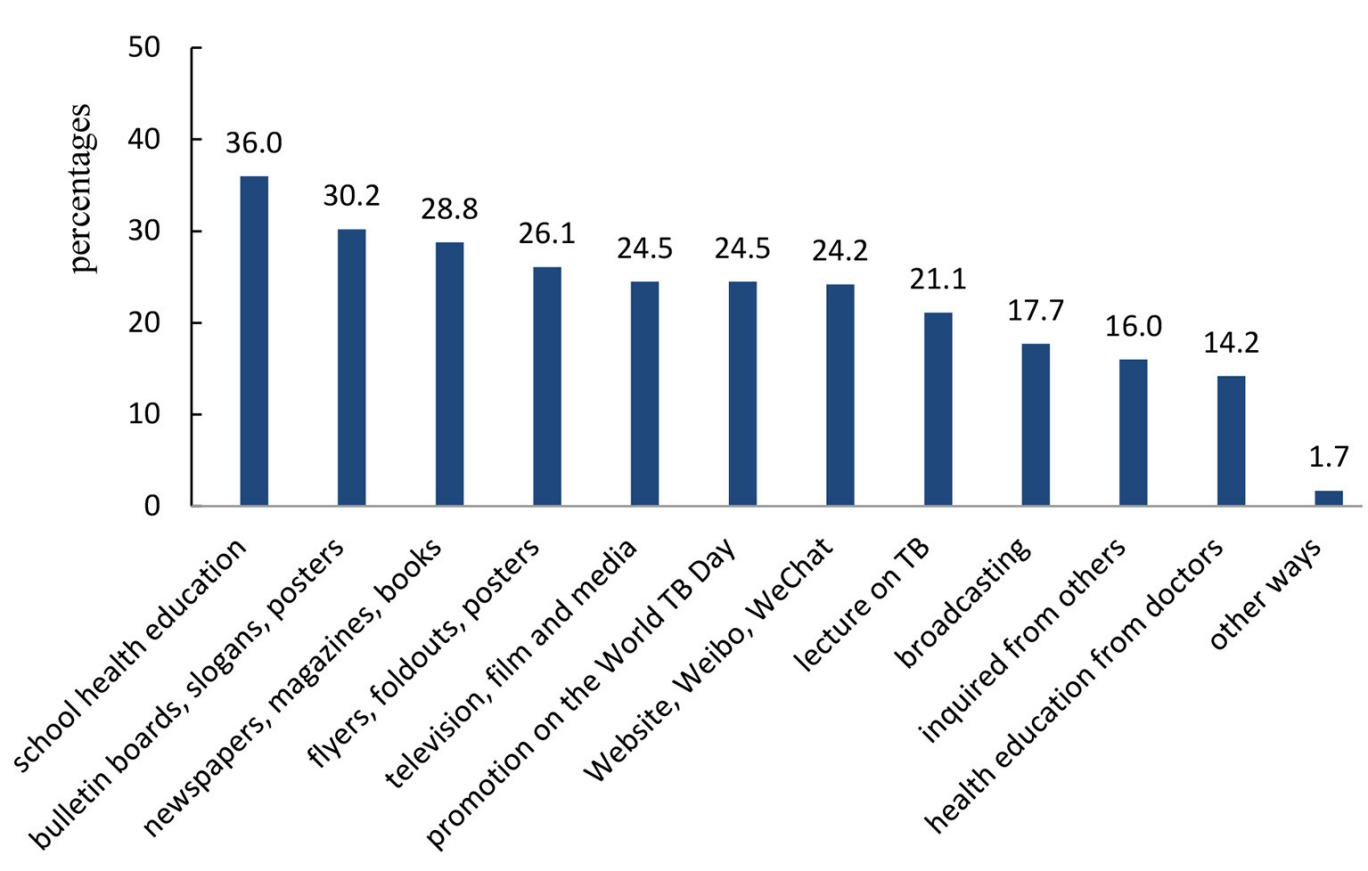
The ways of obtaining the knowledge of TB.

Thirty-six percent, 30.2%, and 28.8% of freshmen claim to obtain knowledge on TB through “school health education,” “publicity boards, slogans, posters,” and “newspapers, magazines, books,” respectively (Figure 1).

### The Related Attitudes Toward TB

Table 5 shows that the attitudes toward the knowledge of TB. It shows that 96.4 and 96.9% of freshmen are willing to learn and tell others about the knowledge of TB, respectively. Regarding attitudes toward classmates who had suffered TB, 39.5% of freshmen chose “be more caring and considerate of him or her,” and 11.7% of them chose “don't know what to do”.

**Table 5:** The related attitudes toward TB (*n* = 583).

**Problem**	**The number of people who chose it (n)**	**Composition ratio (%)**
1. Are you willing to learn about the knowledge of TB?		
Willing	562	96.4
Unwilling	21	3.6
2. If your classmate had suffered TB, how would you treat it?		
Do not communicate with him/her	13	2.2
Try to keep a distance	165	28.3
As usual	107	18.3
Be more caring and considerate of him/her	230	39.5
Don't know what to do	68	11.7
3. Are you willing to tell others what you have learned about TB?		
Willing	565	96.9
Unwilling	18	3.1

### The Demands for the Ways of TB Health Education

Table 6 shows that 63.5, 55.6, and 53.2% of the students were willing to receive health education on TB through “Website, Weibo, WeChat,” “TB Day Publicity Campaign,” and “Publicity Columns, Slogans, and Posters”, respectively. Regarding the presentation of online health education, 66.4 and 64% of freshmen preferred presentations that comprised of “text + video” and “text + picture,” respectively.

**Table 6:** The willingness of freshmen to accept different ways of TB health education (*n* = 583).

**Contents**	**The number of people who accepted it(*n*)**	**Composition ratio(%)**
1. Ways of TB health education		
Broadcasting	229	39.3
newspapers, magazines, newspapers	240	41.2
flyers, brochures, posters	282	48.4
TV, film and media	255	43.7
websites, Weibo, WeChat	370	63.5
bulletin boards, slogans, posters	310	53.2
publicity activities on the day of TB	324	55.6
health education organized by teachers	260	44.6
Lectures about TB	243	41.7
others	30	5.1
2. The Way that online health education		
text + picture	373	64.0
text + link	129	22.1
text + video	387	66.4
animation or presentation	338	58.0
others	24	4.1

The willingness of accepted (%) = ∑ the number of people who accepted the content/583^*^100%.

## Discussion

Tuberculosis health education is one of the TB control strategies in China. The goal is to raise the awareness of TB prevention and treatment policies and knowledge among different groups of people. This is so that they can adopt appropriate behaviors, ultimately implement TB control strategies, and achieve the goal of controlling the TB epidemic. Due to the differences in the needs of TB prevention and treatment, the role that playing, and the benefits and ability of acceptance between different groups of people, it is necessary to carry out targeted TB health education. College students are currently amidst the digital age. Hence, surfing the internet has become their main daily activity (17). Consequently, they not only have a strong understanding and acceptance of knowledge, but they can also spread the knowledge of TB to families and communities through the chain “teacher-student-parent-community”. By extension, this provides a great impact on improving the awareness of TB in the whole society. Therefore, to assess the level of knowledge, attitude, and practice (KAP) on TB among college students has become a common focus of research (18–22). The TB prevention and control among college freshmen cannot be ignored, but the related study is scarce (23). In this study, the relevant baseline data was obtained by assessing the cognitive attitude and health education needs of TB among freshmen so as to provide a reference for implementing policy accurately.

The results of this survey showed that the cognition about the five related knowledge of TB among freshmen was unbalanced. There are some incorrect cognitions on TB among freshmen. Some freshmen believe that TB is an acute infectious disease like other common respiratory diseases, while some are unsure of how long the suspicious symptoms of cough and sputum should last and uncertain about whether TB can be cured. Despite this, there is a high level of cognition about the transmission route, the awareness of active consultation after illness, and the routine preventive measures of TB among freshmen (21–24). The overall awareness rate about the eight core knowledge of TB among freshmen is 73.3%, which is lower than the target (≥85%) among populations as proposed by the “13th Five-Year” National TB Control Plan in China.

The result of the *t-*test reveal that the awareness rate of TB among freshmen is related to the TB health education they received before enrollment and has nothing to do with their basic demographic characteristics. Freshmen with a higher overall awareness rate on the eight core knowledge of TB are those who had suffered TB or had received TB health education before enrollment. However, the breadth and depth of TB education that freshmen have received before enrollment is limited. Hence, the TB health education among college freshmen should be strengthened. Freshmen who have not heard of TB and have not received TB health education before enrollment are key populations in TB health education in colleges. It is necessary to strengthen the theoretical and practical health education on TB from the initial entry of freshmen in colleges.

The study shows that freshmen have a willingness rate of equal or >96%, proving that they are willing to learn and spread the knowledge of TB. However, the passive way of learning and spreading is a limitation as they may be more prone to treat patients with TB in a discriminatory way. Hence, the attitude toward learning and spreading the knowledge of TB among freshmen still needs guidance.

Nowadays, college students are the most active internet user group on campus. Hence, mobile phones and computers have become the main ways for them to socialize and acquire knowledge, a distinctive characteristics of the times. This explains how college students prefer online education platforms with vivid themes, such as “website, Weibo, WeChat”, etc. Most studies also show that the internet has gradually become a new health education tool in colleges as it effectively enriches students' health knowledge and provides an avenue to spread the knowledge of TB to their community. Therefore, actively exploring the Internet + health education form is an inevitable trend to expand and optimize TB health education, which is suitable for the behavioral characteristics of college students.

## Recommendations

There are three suggestions being proposed to optimize TB health education in colleges. Firstly, to combine the centralized health education on “World TB Day” on March 24 each year with the daily regular health education, and to extensively publicize the core knowledge of TB on campus. Secondly, to establish a health education model of TB based on peer education, special lectures, and prescriptions of health education. We also recommend relying on new online media platforms that adapt to the preference of college students in order to optimize the organic combination of interpersonal communication and mass communication while evaluating the effect of TB health education (25–27). Thirdly, to carry out comprehensive and systematic health education of TB prevention and treatment theory and practice, emphasize the unity of knowledge and action, and continuously improve the health literacy level of TB prevention and treatment among college students nowadays.

## Conclusions

In summary, improving the cognition level of the knowledge of TB among college students is crucial. Nowadays, the Internet era has brought new opportunities and challenges to TB health education in colleges. The new finding in the study is that the experience and education on TB before entering college are important. Therefore, the TB health education among students should be carried out in all learning stages continuously. In the college campuses, TB health education should be carried out from the students' initial entry to the campus and be adapted to the behavioral characteristics of freshmen. In addition, it should pay more attention to monitor and evaluate the effect of health education timely.

## Data Availability Statement

The original contributions presented in the study are included in the article/supplementary material, further inquiries can be directed to the corresponding authors.

## Ethics Statement

The studies involving human participants were reviewed and approved by the Research Ethics Board of Health (REBH), Guangxi Medical University, China. The patients/participants provided their written informed consent to participate in this study.

## Author Contributions

TW and QF: conceptualization and resources. TW and SW: data curation and visualization. TW, SW, and QF: formal analysis, validation, writing–original draft, and writing–review and editing. TW, JP, JY, SH, SG, and CY: investigation. TW: methodology. TW, HHu, ZT, HHe, and QF: project administration. All authors contributed to the article and approved the submitted version.

## Funding

This study was supported by Youth Science Foundation of Guangxi Medical University (Project No.: GXMUYSF201831).

## Conflict of Interest

The authors declare that the research was conducted in the absence of any commercial or financial relationships that could be construed as a potential conflict of interest.

## Publisher's Note

All claims expressed in this article are solely those of the authors and do not necessarily represent those of their affiliated organizations, or those of the publisher, the editors and the reviewers. Any product that may be evaluated in this article, or claim that may be made by its manufacturer, is not guaranteed or endorsed by the publisher.
